# Prehabilitation in elective surgical interventions – what must the general and abdominal surgeon know

**DOI:** 10.1515/iss-2023-0006

**Published:** 2023-08-22

**Authors:** Carl Meißner, Frank Meyer, Karsten Ridwelski

**Affiliations:** MVZ “Im Altstadtquartier”, General Surgery, Magdeburg, Germany; Department of General and Abdominal Surgery, Municipal Hospital (“Klinikum Magdeburg gGmbH”), Magdeburg, Germany; Otto-von-Guericke University at Magdeburg, Institute for Quality Assurance in Operative Medicine, Magdeburg, Germany; Department of General, Abdominal, Vascular and Transplant Surgery, Otto-von-Guericke University at Magdeburg with University Hospital, Magdeburg, Germany

**Keywords:** abdominal surgery, enhanced recovery after surgery (ERAS), general surgery, prehabilitation, preoperative risk assessment, supplementation

## Abstract

**Objectives:**

For years, many efforts have been invested to prepare patients, in particular, those with reduced physical and psychic status, much better to provide and finally achieve better outocme if there is time available to provide several beneficial measures.

**Methods:**

Therefore, the objective was to illustrate the concept and various single elements of a complex prehabilitation concept based on (i) selective references from the medical literature and (ii) own clinical experiences from clinical practice in general and abdominal surgery.

**Results:**

Prehabiliation can be considered the solution of the efforts to improve preoperative status for patients in a disadvantageous status for almost all types of surgery and all other operative and/or interventional procedures. It is the targeted process to improve individual functionality and organ function before a planned (elective) surgical intervention; P. comprises basically nutritional, physical and psychological measures; P. focusses especially onto the elderly, frail and malnourished patients before a planned surgical intervention; the overall aim is to significantly improve final outcome characterized by shorter length of stay, lower complication rate and mortality as well as cost efficiency; P. is especially important in cancer surgery, in which the beneficial effects can be particularly implemented; P. programs and/or “Standard Operating Protocols“ (SOP) may help to establish and materialize its single aspects and enhanced recovery after surgery (ERAS). There is still further potential to reliably establish and to utilize the options of prehabilitation measures as listed above.

**Conclusions:**

Prehabiliation is an indispensable aspect in today’s preparation for elective surgery, which needs to become obligatory part of the preparation measures to planned surgical interventions, which can further contribute to a better final outcome and ERAS as well as, in addtion, needs to be further developed and accomplished.

## Introduction

Prehabiliation is the targeted process to improve individual functionality before a planned (elective) surgical intervention and has only been researched for a few years. While at PubMed^®^ under the keyword “Prehabilitation” until 2010 less than ten publications had been published internationally each year, this number had rised to 191 jobs in 2019 and was already at 231 at the end of October 2020. It is about patient – especially elderly, frail and malnourished patients – for a scheduled surgery to strengthen them recover faster and better afterwards. In the Guidelines of the German Society for Nutritional Medicine (DGEM) and the European Society for Clinical Nutrition and Metabolism (ESPEN), in particular, appropriate recommendations for the preoperative phase of surgery related to clinical nutrition are provided. Many patients, who underwent the aspects and recommendations of this concept, experienced enhanced recovery after surgery (ERAS) – various programs also include meanwhile such recommendations.

So far, not every study has been able to confirm the success of previous favorable results achieved in a single study. On the other hand, in many areas of surgery, promising results are already available, such as preoperative ones. Supported by means of oral food supplements (ONS), including medical drinking food, the postoperative recovery could be positively influenced, for example in entities, such as colo-rectal, gastrointestinal and gynecological carcinoma, or in surgical interventions, such as gastrectomy and surgery of the lower gastrointestinal tract.

But also using prehablitation program aspects, including physical exercises, breathing training and mental support could achieve positive results, for example in knee and hip operations in the elderly, heart operations, bronchial carcinoma and chronic obstructive pulmonary disease. Often, combination of different pre-qualification approaches resulted in success.

The aim of this work was to illustrate the concept and various single elements of a compact prehabilitation program recommended and confirmed by scientific studies and reports including suggested aspects not everywhere established in daily work based on selective references from the medical literature and own clinical experiences from clinical practice in general and abdominal surgery.

## Methods

Narrative compact overview on the topic using the following terms for selective literature search: Prehabilitation, general surgery, abdominal surgery, preoperative risk assessment, supplementation, enhanced recovery after surgery (ERAS).

## Results

### The concept of prehabilitation

Larger surgical interventions can be usually considered a serious health burden, especially in older patients with bad cardiorespiratory and nutritional reserves [1]. As stressors, surgical interventions apply anesthesia, intraoperative blood loss, perioperative tissue injury and subsequent systemic inflammatory reactions [2]. Data from Great Britain provide an exemplary impression on the situation [3]:

– The English health system leads about 2,414 major surgical procedures annually per 100,000 inhabitants.
– The mortality of surgical interventions is increasing up to 4 % estimated, with increasing postoperative complications as seen as the main cause.
– A total of 15–40 % of the patients show postoperative complications.
– Whose immediate effects are significant, including a two to four-fold increase in hospital length of stay and increased readmission rate.

### Paradigm shift in nursing after surgical interventions

Rehabilitation is traditionally seen as the mainstay of recovery after surgery [4]. In addition, however, it has been shown that a bad preoperative physical performance, can increase mortality, the probability and number of postoperative complications as well as can delay postoperative recovery [5–7], in particular, due to factors such as poor physical function, poor respiratory muscle strength, smoking, alcohol abuse, under- or overeating, and poor mental health.

As a following concequence, reduced physical constitution increases the risk of delayed rehabilitation [3].

The preoperative nutritional status plays a central role in old age, especially in cancer. Malnutrition can increase the risk, especially after major surgery for complications in the further postoperative course [8]. Furthermore, it favors sarcopenia (the progressive degeneration of skeletal muscle mass) and cachexia (extreme weight loss and muscle wasting due to chronic illness) and impairs the quality of life in cancer patients [9]. A recent study found that nearly two thirds of those elderly patients who had been studied in the hospital experienced a tissue loss syndrome (i.e., sarcopenia, frailty, cachexia, or malnutrition) [10]. This is worrying since, for example, sarcopenia alters 1-year mortality rate of older people with cancer [11]. The increase in overweight and obesity in the Western world can also result in the occurrence of malnutrition, which is, then, rarely realized. And, a disease-associated weight loss affects in particluar, especially overweight patients not necessarily leading to the WHO’s definition of body mass index (BMI) for malnutrition of less than 18.5 kg/m^2^. A weight loss (in) itself means a “metabolism one risk” due to the change in body composition that – particularly – occurs in patients with major surgcial interventions, especially tumor operations, which must be taken into account [8, 12].

The concept of “pre-qualification“ therefore provides the best possible state of health and the individually optimized functionality of the single patient preoperatively. These patients have better reserves after an operation. The amount of functional loss has also a significant impact onto the physical condition before the surgcial intervention [2].

### Definition of prehabiliation

Prehabiliation refers to the process of improving individual functionality before a planned surgical procedure that aims to improve the patient’s tolerance to impending physiological stress [4].

## Prehabilitation=get fit for the operation!

Prehabilitation has been relatively well established in the cardiovascular and thoracic surgery [13–15]. It is one tool for our patients [16]. In contrast, in older patients who are scheduled for abdominal surgery, including oncosurgery, it is less extensively spread. In case of these patients, the nutritional status before the surgical intervention is particularly important to improve and counteract muscle wasting. Recently, it had been made available to patients who had to undergo colorectal tumor resection: A “trimodal” multidisciplinary preoperative program was initiated to improve (i) physical condition, (ii) nutritional status and (iii) the preoperative anxiety that contains moderate physical activity, nutritional advice, and protein supplementation as well as coping strategies [17].

### Scope of prehabilitation

Prehabilitation is considered an interdisciplinary process and essentially comprises according to Know’s rules [2]:

– Exercise and physical therapy,
– Targeted support through enteral nutrition (and)
– Management of preoperative anxiety and psychological support.

By further flattening hospital hierarchy, interdisciplinary communication between the specific departments, increased patient centricity and role enhancement of interdisciplinary,-professional and-sectional team members (patients and their family members, physicians, nurses, other health workers) can further promote this process [18].

#### Pre-operative physical exercises and physiotherapy

Patients should be brought the best physical condition possible. The special ones proposed for a training program (physiotherapy, exercise and sports therapy) according to Bloch aim at [19]:

– Musculoskeletal system,
– Cardiovascular system,
– Lung function,
– Metabolism (and)
– Immune system.

The preoperative physical exercises should be adapted to the specific patient’s condition and include endurance, strength and coordination training of various intensities. Usually, the spectrum ranges from simple gymnastic exercises and training on the bicycle ergometer via strength training with and without special equipment to training in the water, sensory-motor training or respiratory muscle training. This not only activates the muscles and cardiovascular system, but also other body functions, such as joint function and immune system [19]. In a current systematic review on the influence of preoperative physical activity on the postoperative course of knee and hip replacement, operations in older patients demonstrated that most of the studies on knee surgery show a trend for improvement of the visual analog scale (VAS) of the range of motion (ROM), functional scores (such as Knee injuries and Osteoarthritis Outcome Score [KOOS] and Western Ontario McMaster University Osteoarthritis Index [WO MAC]) and quality of life (Short Form-36 [SF-36]) as well as shorter length of stay (one of the secondary study parameters). After hip surgery, preoperative activity led to an improved postoperative functional recovery (among primary study parameters) [20]. In another review, the effect of preoperative physical exercises for heart surgery was examined. The available studies showed that the exercise groups showed a significantly shorter stay on the intensive care unit *versus* (*vs*.) the control groups as well as better postoperative physical functional recovery [21].

In a meta-analysis, the effect of pre-operative exercise and breathing therapy was studied demonstrated by the surgical results in patients with lung cancer and chronic obstructive pulmonary disease (COPD): Patients who received preoperative training showed a lower incidence of postoperative pulmonary complications and a shorter hospital stay. In patients with COPD, no fewer postoperative complications, but a faster recovery occurred [22, 23].

#### Pre-operative support through targeted nutrition

Nutritional support is a key aspect of perioperative management [8, 12] and is an increasing integral part of many surgical programs worldwide [2]. A sufficient supply of nutrients in the preoperative phase is vital, not only related to the catabolic effects of the underlying disease and surgical intervention to counteract stress, but also to promote the tissue healing process after surgery [24].

In particular, an adequate protein supply plays an important role, as it provides the basis for supplying the muscles with amino acids [25]. Aminoacids not only stimulate the synthesis of structural proteins, such as myofibrillar proteins, but also the synthesis of mitochondrial proteins, which are essential for aerobic metabolism and the maintenance of functional training capacity as necessary [26]. This stimulating effect is achieved by an increased recovery process as promoted by athletic physical exercise [27].

A clinical study of 102 patients with gynecological or gastrointestinal tumor lesions treated according to ERAS guidelines found that 14 days of supplementation with a high-protein medical sip food before the operation led to a smaller number of serious postoperative complications [28].

Another study looked at the effect of a perioperative drinking food administration in 127 undernourished patients after gastrectomy [29]. While the complication rate in the overall collective of both groups was not significantly different, the use of medical sip food in severely undernourished patients (grade C) showed a significant improvement in the form of a lower incidence (all complications), duration (complications over 3 or 5 weeks postoperatively) as well as severity of complications (grade, ≥ IIIa).

A further study came to similar results in 152 patients who underwent surgery at the lower gastrointestinal (GI) tract [30]. The patients who had preoperatively received a medicated sip food showed a significantly decreased weight loss postoperatively compared to those without food supplements as well as a lower incidence of minor complications. Furthermore, the average total costs in these patients were lower.

#### Pre-operative anxiety management and psychological support

The phase before an operation can be very frightened for many patients, especially in elderly patients. Studies show that fear and depression often affect the postoperative outcomes characterized by longer hospital stays, higher risks of infection and a poorer recovery of the patient [31–33]. Thats why, it is important to such threatened patient to receive psychosocial support in the preoperative time period.

In many pre-qualification programs, medical personnel, such as nurse, clinician or psychologist, is involved to help the elderly surgical patients with regard to emotional and psychological support. Indeed, it has been shown that a 60 min visit to a trained psychologist and certain home therapies, such as relaxation with breathing exercises, were able to help reducing the patient’s anxiety [17].

Emotional and psychological supports are therefore an important part of pre-qualification and can also contribute to adhere and motivate patients to nutritional standards and exercise therapy [2].

### Guidelines related to preoperative clinical nutrition

#### DGEM S3 guideline on clinical nutrition in surgery

In 2013, the existing guidelines of the German Society for Nutritional Medicine (DGEM) and the ESPEN on enteral and parenteral nutrition were merged, updated and expanded [8]. Before the background of existing ERAS programs as standard in perioperative management, the present S3-guideline formulates 41 evidence-based recommendations for the use of “artificial nutrition” (oral/enteral and/or parenteral) for surgical risk patients, for major tumor operations (as well as) if severe complications occur.

With regard to preoperative nutrition, the DGEM in principle recommends that patients with a severe metabolic risk1Surgical patients are at severe metabolic risk if at least one of the following criteria is met: **a.** Weight loss over 10–15 % within 6 months; **b.** BMI below 18.5 kg/m^2^; **c.** Subjective Global Assessment (SGA) Grade C or Nutritional Risk Screening (NRS 2002) greater than 3; **d.** Serum albumin below 30 g/L if liver or kidney dysfunction can be ruled out preoperatively. before the operation should receive nutritional therapy if the surgical intervention needs to be postponed (Recommendation 16). Further recommendations are (Recommendations 17 to 22):

– Whenever possible and feasible, it should be fed orally.
– Because many patients have their energy needs in the preoperative phase, the supply may not always adequately cover the necessary diet, regardless of the nutritional status as provided by sip food.
– Malnourished cancer patients and such at high risk should be given hydration nutrition prior to major abdominal surgery.
– Immunomodulating diets (with arginine, omega [Ω]-3 fatty acids and nucleotides) are to be favoured.
– A preoperative supplementation with drinking food or enteral feeding should be preferably given before hospital admission started in order not to unnecessarily extend the hospital stay and reduce the risk for nosocomial infection.
– A preoperative parenteral nutrition should be administered in patients with severe metabolic risks when an adequate supply of energy via enteral administration cannot be guaranteed.

#### ESPEN guidelines for clinical nutrition in surgery

In 2017, the European Society for Clinical Nutrition and Metabolism (ESPEN) published its updated guidelines on clinical nutrition in surgery [34]. These guidelines were derived from the ESPEN guideline for enteral nutrition: surgery and transplantation [35], ESPEN guideline for parenteral diet: surgery [36] as well as DGEM guideline on clinical nutrition in surgery [8].

As the DGEM guideline from 2013, the ESPEN guideline based on the nutritional aspects of the ERAS concept and the special nutritional needs of patients undergoing major surgery, e.g., in cancer, as well as of those patients who develop severe complications despite the best perioperative care. The ESPEN guideline formulates 37 recommendations.

Comparable to the recommendations of the DGEM, according to ESPEN, preoperative nutritional support makes sense under the following aspects (Recommendations 14 to 20) [34]:

– Patients at severe metabolic risk2Same criteria as DGEM [8], except for Nutritional Risk Screening (NRS 2002) over 5 (instead of 3). should receive nutritional therapy before a major surgical intervention, even if the operation has to be postponed – even if the patient has a malignant tumor lesion (recommended time period ranging from 7 to 14 days).
– Whenever possible, use the oral/enteral route preferentially.
– When patients are unable to meet their energy needs covered by normal food, it is recommended to encourage patients to take oral food supplements before surgery, regardless of their nutritional status.
– Pre-operatively, all malnourished cancer and high-risk patients who are planned to undergo major abdominal surgery, should be given oral food supplements. A special group of high-risk patients are elderly people with sarcopenia.
– Immunomodulating oral food supplements with arginine, Ω−3 fatty acids and nucleotides can be preferred and can be preoperatively administered for 5 up to 7 days.
– Preoperative enteral nutrition/oral nutritional supplements should preferably be given before hospitalization in order to avoid unnecessary hospital stays to reduce the risk of nosocomial infections.
– Preoperative parenteral nutrition is allowed only in patients with malnutrition or severe nutritional risk whose energy requirements cannot be adequately covered by enteral nutrition. A time period of 7–14 days should be considered for this recommendation before surgery.

#### ERAS guidelines for nutritional prehabilitation

Enhanced recovery after surgery (ERAS) is a recovery phase improvement concept after surgical interventions associated with a reduction in the complication rate, shortening hospital stay and reduction of the associated costs [8]. The ERAS programs have now become an international standard in perioperative management of many surgical specialties, which were appropriately and subject-specifically adopted [34].

In addition to subject-specific recommendations, many ERAS programs also contain recommendations for nutritional prehabilitation (see Table 1). Other ERAS programs have dealt with this option not yet. These include cyto-reductive surgery [37], total hip and knee replacement [38], surgery of the newborn bowel [39], Caesarean section [40], breast reconstruction [41] and surgery for severe head and neck cancer [42]. Many other preoperative recommendations out of ERAS programs relate to quitting habitual smoking and drinking harmful amounts of alcohol, weight loss for obesity (as well as) management of fasting immediately before surgical intervention and filling of carbohydrate stores. Enteral and/or parenteral nutrition are also part of the postoperative management in ERAS programs.

**Table 1:** ERAS recommendations for nutritional prehabilitation.

ERAS recommendation for nutritive prehabilitation	Degree of recommendation	Evidence base/level
Colorectal surgery [43]		

Routine nutritional screening for detection and correction from malnutrition	Severely	Low
Oral nutritional support for patients at risk for malnutrition for at least 7–10 days	Severely	Moderate

Gastrectomy [44]		

Oral food supplements or enteral nutrition for significantly malnourished patients	Strong	Very low
No routine use of preoperative artificial nutrition	Strong	Very low

Esophagectomy [45]		

Nutritional screening to identify and optimize the nutritional status	Strong	Low
Enteral support preferably via the GI tract with selective use of feeding tubes in high-risk cases	Strong	Low

Head and neck cancer surgery [42]		

Comprehensive nutritional screening with special attention on dysphagia and refeeding syndrome	Strong	High
Nutritional intervention for malnourished patients	Strongly high	
Use of standard polymeric enteral formula for patients, needing nutritional support	Weak	Low

Lung surgery [46]		

Screening for nutritional status and weight loss	Strong	High
Oral food supplements for high-risk patients as a supplement to total intake	Strong	Moderate

Cardiac surgery [47]		

Correction of nutrient deficiency if feasible	Moderate	Low
Prehabiliation (through counseling, nutritional optimization, exercise training, social support and reduction of fears) for patients who want to undergo elective surgery, with multiple comorbidities or exhibit significant deconditioning	Strong	Moderate

Liver surgery [48]		

Oral food supplements for risk patients (weight loss over 10 up to 15 % within 6 months, BMI below 18.5 kg/m^2^ & serum albumin below 30 g/L in the absence of liver or kidney dysfunction)	Strong	High
To postpone the operation for at least 2 weeks in severely under-fed patients (more than 10 % weight loss) for improvement nutritional status and allowing weight gain	Strong	High

Bariatric surgery [49]		

Preoperative weight loss (risk of hypoglycaemia in patients with glucose-lowering drugs)	Strong	Very high^a^ Low^b^

Gynecological oncology [50]		

Certain patients benefit clinically from prehabilitation, but further studies in gynecological oncology are needed	Weak	Low

Radical cystectomy for bladder cancer [51]		

Nutritional support, especially high in malnutrition	Strong	High

Rectal and pelvic surgery [52]		

Special foods for malnourished patients	Strong	Moderate

^a^Related to postoperative complications. ^b^Related to postoperative weight loss.

### Prehabiliation for colorectal cancer

Colon cancer is the second most common cancer in women and the third most common in men worldwide [53]. In an international comparison, Germany is one of the countries with a particularly high incidence, which was 57.0 in 2012 per 100,000 for men and 36.5 per 100,000 for women, with a mortality of 23.5 men per 100,000 and 14.4 per 100,000 women [54]. Especially older patients with colorectal cancer after surgical intervention experience, among other things, postoperative complications, major weight loss, extended hospital stays and a high recovery rate associated with high economic costs [55].

Prehabilitation is one tool in the therapy of sugery in colorectal caner [56].

In a systematic review and meta-analysis, the effect of nutrition-based and multimodal pre-qualification programs was studied in colorectal operations [29]. Studies were included, in which the patients received nutritional support for at least 7 days preoperatively, with or without an exercise program. The primary parameter was length of hospital stay, secondary parameter was the restoration of the functionality based on the results obtained in a 6-min walking distance test (6 MWD). A total of 9 studies (5 randomized controlled trials and 4 cohort studies) were identified with 914 patients (438 received pre-qualification measures and 476 served as controls):

Prehabilitation overall led to a significant reduction in the length of hospital stays compared to controls by 2.2 days (95 % confidence interval [CI], −3.5 to −0.9). Due to great methodical differences between the studies, the multimodal pre-rehabilitation approach can only assume that the results of the 6-MWD test will also have improved after 4 and 8 weeks.

In another study, the effect by a trimodal prehabilitation – in the form of physical, nutritional and psychosocial exercises – on the cumulative 5-year [yr] disease-free survival rate (5-yr-DFS) and the 5-yr-overall survival rate (5-yr-OS) after colon cancer surgery was investigated [57]. For that, data was collected from two randomized clinical studies and a cohort study resulting in a total of 202 patients, 104 in the pre-qualification group and 98 in the control group. The median prehabilitation time was 29 days.

While in this study, the 5-yr-OS showed no difference between the groups, the 5-yr-DSF in the pre-qualification group for level-III patients was significantly higher than in the control group (73.4 % vs. 50.9 %; p=0.044). When all patients in stages I to III were combined, the differences were no longer significant. Furthermore, pre-qualification was found, after adjustment for cancer stage and other confounding factors, as independent prediction factor for improved 5-yr-DFS (hazard ratio, 0.45; 95 % CI, 0.21–0.93), but not for OS.

## Summary

Prehabiliation in the sense of targeted preoperative support by means of (additional) nutrition and physical exercise and psychological/psychotherapeutic measures are increasingly finding their way into elective surgery, especially in the elderly, frail and malnourished patients. Many new studies indicate that through prehabilitation, the nutritional status, the length of stay in the clinic, the complication rates and cost efficiency can be significantly improved.

In our own experience, pre-habilitation is a good way to provide patients with comprehensive care. The compliance, especially in programs, is very good and the current guidelines support the procedure.

Nutritional support in the form of medicinal sip food (also known as ONS – Oral Food Supplements) plays a central role in prehabilitation and is already recommended in most ERAS programs. The guidelines of DGEM and ESPEN also underline their importance to patients regardless of their nutritional status. On principle, ONS should be given to all malnourished cancer and high-risk patients, who are planned to undergo major abdominal surgery.

Looking ahead, it should be more focussed on such preoperative measures to ensure the success of prehabilitation programs, which take a number of factors into account. Among other things, the motivation of the patients plays an essential role, continuously “To stay on the ball” [2]. In this context, the number of supervising nursing staff in the clinics must be sufficient to carry out preoperative measures such as movement exercises. This also applies to patients who prefer to be at home before the surgical intervention wanting to stay. It would also be desirable to develop technical tools and “apps” for instruction and monitoring of prehabiliation programs or corresponding video programs in real-time transmission.

In principle, further studies with a uniform structure performing the positive effect of prehabilitation programs – including nutritional support – on conservation and recovery of the physical and mental health after elective surgical interventions are welcome to explore the consequences of operations further.

In summary, there is still further potential to reliably establish and to utilize the options of prehabilitation measures as listed above.

In conclusion, prehabiliation is an indispensable aspect in today’s preparation for elective surgery, which needs to become obligatory part of the preparation measures to planned surgical interventions, which can further contribute to a better final outcome and ERAS as well as, in addtion, needs to be further developed and accomplished.

## Supplementary Material

Supplementary Material
