# Supplementary material for: Prehabilitation in elective surgical interventions – what must the general and abdominal surgeon know
Source: Innov Surg Sci. 2023 Aug 22;8(2):93–101. doi: 10.1515/iss-2023-0006 (PMC10696941; doi:10.1515/iss-2023-0006)
Supplement: Supplementary file 1 — Supplementary Material [file j_iss-2023-0006_suppl_001.pdf]

Reviewer Assessment

C. Meißner, F. Meyer & K. Ridwelski: Prehabilitation in elective surgical interventions - what must the general and abdominal surgery know

Reviewers' Comments to Original Submission

Reviewer 1: Manger, Thomas

Date received: 21-Feb-2023
Reviewer recommendation: **Accept in present form**
Reviewer overall scoring: **Medium**

Comments to author:
Thanks for the interesting overview. Please check the grammar.

Reviewer 2: anonymous

Date received: 23-Feb-2023
Reviewer recommendation: **Return to author for major modifications**
Reviewer overall scoring: **Medium**

Comments to author:
The authors present an interesting narrative review regarding a topic of high clinical importance. While focusing on nutritional prehabilitation the authors included literature up to October 2020.
-An update of the literature is recommended.
-The review of the DGEM/ESPEN guideline recommendations (p. 15-17) may be reduced.
-It may be interesting whether there is evidence for specific nutritional recommendations like high protein, immunonutrition , probiotics.etc. .
-The own experience may be described more in detail.
-Language editing is recommended: e.g. title:. what must the general and abdominal surgeon know? . pre-qualification = prehabilitation?

Authors' Response to Reviewer Comments

Date received: 14-May-2023

Reviewer 1:

Thanks for the interesting overview. Please check the grammar.

We checked the grammar again.

Reviewer 2:

The authors present an interesting narrative review regarding a topic of high clinical importance. While focusing on nutritional prehabilitation the authors included literature up to October 2020.

-An update of the literature is recommended.

New:

Charlotte Jl Molenaar, Stefan J van Rooijen, Hugo Jp Fokkenrood, Rudi Mh Roumen, Loes Janssen, Gerrit D Slooter, Prehabilitation versus no prehabilitation to improve functional capacity, reduce postoperative complications and improve quality of life in colorectal cancer surgery. Cochrane Database Syst Rev. 2022 May 19;5(5):CD013259.

Gillis C, Ljungqvist O, Carli F. Prehabilitation, enhanced recovery after surgery, or both? A narrative review. Br J Anaesth. 2022 Mar;128(3):434-448. doi: 10.1016/j.bja.2021.12.007. Epub 2022 Jan 7.

Malcolm A West, Sandy Jack, Michael P W Grocott, Prehabilitation before surgery: Is it for all patients? Best Pract Res Clin Anaesthesiol. 2021 Dec;35(4):507-516.

Arved Weimann, Marco Braga, Franco Carli, Takashi Higashiguchi, Martin Hübner, Stanislaw Klek, Alessandro Laviano, Olle Ljungqvist, Dileep N Lobo, Robert G Martindale, Dan Waitzberg, Stephan C Bischoff, Pierre Singer. ESPEN practical guideline: Clinical nutrition in surgery. Clin Nutr. 2021 Jul;40(7):4745-4761. doi: 10.1016/j.clnu.2021.03.031. Epub 2021 Apr 19.

-The review of the DGEM/ESPEN guideline recommendations (p. 15-17) may be reduced.

Was shortened a bit.

-It may b einteresting whether there is evidence for specific nutritional recommendations like high protein, immunonutrition , probiotics.etc. .

That's a very good hint. We left it out here as it was based on post-operative analysis. See abstract.

Daren K Heyland, Jayshil Patel, Danielle Bear, Gordon Sacks, Heidi Nixdorf, Jennifer Dolan, Marianne Aloupis, Kate Licastro, Vera Jovanovic, Todd W Rice, Charlene Compher. The Effect of Higher Protein Dosing in Critically Ill Patients: A Multicenter Registry-Based Randomized Trial: The EFFORT Trial. JPEN J Parenter Enteral Nutr. 2019 Mar;43(3):326-334. doi: 10.1002/jpen.1449. Epub 2018 Sep 27.

Current randomized trials and observational studies evaluating higher versus lower protein doses in critically ill patients yield inconclusive results. Because of few studies and methodologic limitations, clinical guidelines suggest a wide range of protein intake based on weak evidence. Clinical equipoise about protein dosing exists. The purpose of the current manuscript is to provide the rationale and protocol for a randomized controlled trial (RCT) of 4000 critically ill patients randomly allocated to receive a higher or lower protein dose. We propose a global, volunteer-driven, registry-based RCT involving >100 intensive care units (ICUs). We will enroll mechanically ventilated patients with high nutrition risk, identified by low (≤25) or high (≥35) body mass index, moderate to severe malnutrition, frailty, sarcopenia, or when >96-hour duration of mechanical ventilation is expected. Exclusion criteria include patients who are >96 hours since initiation of mechanical ventilation, moribund, or pregnant, and where the clinician lacks clinical equipoise regarding protein dose. The intervention consists of higher (≥2.2 g/kg/d) or lower (≤1.2 g/kg/d) protein dose, achieved by enteral nutrition, parenteral nutrition, or both. The primary outcome will be 60-day mortality. Key secondary outcomes include time-to-discharge alive from hospital, ICU and hospital survival, and length of stay. As this is research based on existing medical practice, we will apply for a waiver of informed consent, where possible. The large sample size is a reflection of the small signal we expect to see in this large, pragmatic trial.

-The own experience may be described more in detail.

We included that in the summary.

-Language editing is recommended: e.g. title:. what must the general and abdominal surgeon know? . pre-qualification = prehabilitation?

Prehabilitation.

Reviewers’ Comments to Revised Submission

Reviewer 1: Manger, Thomas

Date received: 18-May-2023
Reviewer recommendation: **Accept in present form**
Reviewer overall scoring: **High**

Comments to author:

Dear Author, the paper does now fulfil the suggestions for publication.

Reviewer 2: anonymous

Date received: 29-May-2023
Reviewer recommendation: **Return to author for minor modifications**
Reviewer overall scoring: **Medium**

Comments to author:

The authors considerably improved the manuscript.

Some more minor suggestions:
p. 16 omit In 2013 ... until p. 17 cannot be guaranteed
p. 17 line 58 and 59 Surgical patients are at severe metabolic risk … shift to p. 18 line 55
p. 20 line 17 instead of (Weimann et al, 2013). Ljungqvist et al, 2017
Ljungqvist O, Scott M, Fearon KC. Enhanced Recovery After Surgery: A Review. JAMA Surg. 2017 Mar 1;152(3):292-298. doi: 10.1001/jamasurg.2016.4952. PMID: 28097305.
p. 21 line 6 better: There is an ongoing debate regarding the need for enteral and parenteral nutrition as part of the postoperative management in ERAS programs for patients undergoing major surgery (Gianotti et al, 2023)
Gianotti L, Paiella S, Frigerio I, Pecorelli N, Capretti G, Sandini M, Bernasconi DP. ERAS with or without supplemental artificial nutrition in open pancreatoduodenectomy for cancer. A multicenter, randomized, open labeled trial (RASTA study protocol). Front Nutr. 2023 Mar 27;10:1113723. doi: 10.3389/fnut.2023.1113723. PMID: 37051129; PMCID: PMC10083279

p. 32 It is recommended to cite only the most recent guidelines

Typos: Abstract: Results: basicly
p. 12 line 6 according to Kow`s rules
p.14 line 6 omit studied, line 53 as promoted
p. 16 line 15 - prehabilitation programs

Authors’ Response to Reviewer Comments on Revised Submission

Date received: 01-Jun-2023

Thank you for the quick and intensive review, as well as the helpful suggestions for improvement. We have tried to incooperate it into the text. Many thanks to the Reviewer 1 and 2.

Comments by the Editorial Office to the Editor-in-Chief Decision

All reviewer comments were addressed adequately and the manuscript should be published in its present stage.
